# (*E*)-2-Eth­oxy-6-[(4-ethoxy­phen­yl)imino­meth­yl]phenol

**DOI:** 10.1107/S1600536810006434

**Published:** 2010-02-24

**Authors:** Arzu Özek, Başak Koşar, Çiğdem Albayrak, Orhan Büyükgüngör

**Affiliations:** a Department of Physics, Ondokuz Mayıs University, TR-55139, Samsun, Turkey; b Faculty of Education, Sinop University, Sinop, Turkey

## Abstract

In the asymmetric unit of the title compound, C_17_H_19_NO_3_, there are three independent mol­ecules, which are align nearly parallel to each other and adopt the phenol-imine tautomeric form. In each mol­ecule, an intra­molecular O—H⋯N hydrogen bond results in the formation of an *S*(6) ring motif. The dihedral angles between the aromatic rings in the three independent mol­ecules are 13.55 (2), 21.24 (2) and 46.26 (1)°. C—H⋯π inter­actions are also observed in the crystal structure.

## Related literature

For related structures, see: Odabaşoğlu, Arslan *et al.* (2007 ▶); Odabaşoğlu, Büyükgüngör *et al.* (2007 ▶); Özek *et al.* (2009 ▶). For details of hydrogen-bond motifs, see: Bernstein *et al.* (1995 ▶).
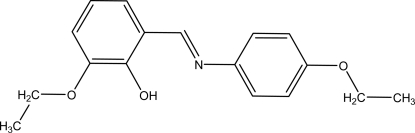


## Experimental

### 

#### Crystal data

C_17_H_19_NO_3_
*M* *_r_* = 285.33
Triclinic, 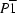

*a* = 11.565 (5) Å
*b* = 14.010 (4) Å
*c* = 15.062 (4) Å
α = 77.229 (4)°
β = 84.398 (5)°
γ = 73.892 (5)°
*V* = 2284.9 (13) Å^3^
*Z* = 6
Mo *K*α radiation
μ = 0.09 mm^−1^
*T* = 296 K
0.72 × 0.34 × 0.12 mm

#### Data collection

Stoe IPDSII diffractometer
Absorption correction: integration (*X-RED32*; Stoe & Cie, 2002 ▶) *T*_min_ = 0.957, *T*_max_ = 0.992
25618 measured reflections
8981 independent reflections
4754 reflections with *I* > 2σ(*I*)
*R*_int_ = 0.042

#### Refinement

*R*[*F* ^2^ > 2σ(*F* ^2^)] = 0.062
*wR*(*F* ^2^) = 0.185
*S* = 1.03
8981 reflections
578 parameters
28 restraints
H atoms treated by a mixture of independent and constrained refinement
Δρ_max_ = 0.45 e Å^−3^
Δρ_min_ = −0.50 e Å^−3^

### 

Data collection: *X-AREA* (Stoe & Cie, 2002 ▶); cell refinement: *X-RED32* (Stoe & Cie, 2002 ▶); data reduction: *X-RED32*; program(s) used to solve structure: *SHELXS97* (Sheldrick, 2008 ▶); program(s) used to refine structure: *SHELXL97* (Sheldrick, 2008 ▶); molecular graphics: *ORTEP-3 for Windows* (Farrugia, 1997 ▶); software used to prepare material for publication: *WinGX* (Farrugia, 1999 ▶).

## Supplementary Material

Crystal structure: contains datablocks I, cigdem3. DOI: 10.1107/S1600536810006434/is2525sup1.cif

Structure factors: contains datablocks I. DOI: 10.1107/S1600536810006434/is2525Isup2.hkl

Additional supplementary materials:  crystallographic information; 3D view; checkCIF report

## Figures and Tables

**Table 1:** Hydrogen-bond geometry (Å, °) *Cg*1, *Cg*2, *Cg*3 and *Cg*4 are the centroids of the C1*B*–C6*B*, C1*C*–C6*C*, C10*A*–C15*A* and C10*C*–C15*C* rings, respectively.

*D*—H⋯*A*	*D*—H	H⋯*A*	*D*⋯*A*	*D*—H⋯*A*
O1*A*—H1*A*⋯N1*A*	0.89 (4)	1.76 (4)	2.601 (3)	156 (4)
O1*B*—H1*B*⋯N1*B*	0.88 (4)	1.80 (4)	2.611 (3)	152 (4)
O1*C*—H1*C*⋯N1*C*	0.92 (4)	1.79 (4)	2.643 (3)	153 (3)
C7*C*—H71*C*⋯*Cg*1	0.97	2.72	3.5692 (1)	146
C7*A*—H72*A*⋯*Cg*2^i^	0.97	2.75	3.6644 (1)	157
C16*B*—H16*G*⋯*Cg*3^ii^	0.97	2.89	3.7935 (1)	156
C16*B*—H16*F*⋯*Cg*4	0.97	2.78	3.6694 (1)	153

Symmetry codes: (i) 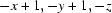; (ii) 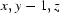.

**Fig. 1.**
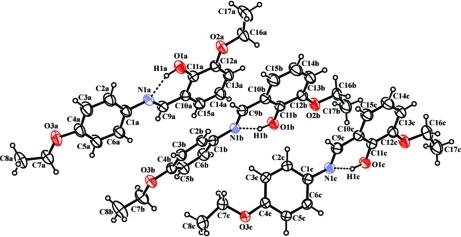
A view of (I), with the atom-numbering scheme and 30% probability displacement ellipsoids. Dashed line indicates intramolecular hydrogen bond.

**Fig. 2.**
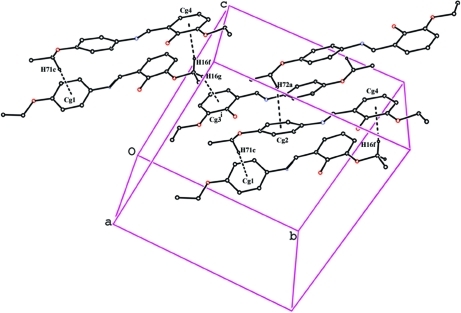
A partial packing view of (I), showing C—H···π interactions. Hydrogen bonds are shown as dashed lines. H atoms not involved in hydrogen bonding have been omitted for clarity. [Symmetry code: (i) 1-x, 1-y, 2-z.]

## supplementary crystallographic information

### Comment

The present work is part of a structural study of Schiff bases (Özek *et al.*, 2009; Odabaşoğlu, Arslan *et al.*, 2007; Odabaşoğlu, Büyükgüngör *et al.*, 2007) and we report here the structure of (*E*)-2-ethoxy-6-[(4-ethoxyphenylimino)methyl]phenol, (I).

In general, *O*-hydroxy Schiff bases exhibit two possible tautomeric forms, the phenol-imine (or benzenoid) and keto-amine (or quinoid) forms. Depending on the tautomers, two types of intra-molecular hydrogen bonds are possible: O—H···N in benzenoid and N—H···O in quinoid tautomers. The H atom in title compound (I) is located on atom O1, thus the phenol-imine tautomer is favored over the keto-amine form, as indicated by the C9—N1, C9—C10, C11—O1 and C10—C11 bond lengths. The O1—C11 bond lengths in molecule A, B and C [1.347 (3), 1.346 (3) and 1.349 (3) Å, respectively] indicate single-bond character, whereas the N1—C9 bond lengths [1.277 (4), 1.274 (4) and 1.279 (3) Å, respectively] indicate a high degree of double-bond character. A similar work was observed for (*E*)-2-[(4-Ethoxyphenyl)iminomethyl]-4-methoxyphenol [C—O = 1.351 (2) Å and C—N = 1.285 (2) Å; Özek *et al.*, 2009].

There are three crystallographic independent molecules A, B and C in the asymmetric unit (Fig. 1) with their ethoxy groups pointing in same directions. The molecular structure of (I), is not planar and this non-planarity increase gradually with the sequence of molecule A, B and C. The dihedral angles between the C1–C6 and C10–C15 benzene rings are 13.55 (2), 21.24 (2) and 46.26 (1)° with this A, B, C sequence. It is known that Schiff bases may exhibit thermochromism or photochromism, depending on the planarity or non-planarity of the molecule, respectively. Therefore, one can expect photochromic properties in (I) caused by non-planarity of the molecules. Intramolecular O—H···N hydrogen bonds result in the formation of a nearly planar six-membered ring motif S(6) (Bernstein *et al.*, 1995), which is oriented with respect to the fused aromatic rings at dihedral angles of 1.22 (1) and 12.38 (1)° for molecule A, 3.28 (1) and 20.28 (1)° for molecule B and 3.26 (1) and 45.49 (1)° for molecule C.

The crystal packing is also stabilized by C—H···π interactions [C7C—H71C···*Cg*1, C7A—H72A···*Cg*2, C16B—H16G···*Cg*3 and C16B—H16F···*Cg*4; Fig. 2 and Table 1). *Cg*1, *Cg*2, *Cg*3 and *Cg*4 are the centroids of the C1B–C6B, C1C–C6C, C10A–C15A and C10C–C15C rings, respectively.

### Experimental

For the preparation of (*E*)-2-ethoxy-6-[(4-ethoxyphenylimino)methyl]phenol compound, the mixture of 3-ethoxy-2-hydroxybenzaldehyde (0.5 g, 3 mmol) in ethanol (20 ml) and 4-ethoxyaniline (0.41 g, 3 mmol) in ethanol (20 ml) was stirred for 1 h under reflux. The crystals suitable for X-ray analysis were obtained from ethanol by slow evaporation (yield 85%; m.p. 363-365 K).

### Refinement

Atoms H1A, H1B and H1C were located in a difference map and refined isotropically. The remaining H atoms were positioned geometrically [C—H = 0.93 Å (aromatic) and C—H = 0.96 Å (methyl)] and constrained to ride on their parent atom, with *U*_iso_(H) = x*U*_eq_(C), where x = 1.5 for methyl H, and x = 1.2 for other H atoms. To recover the slightly deformed shape of the ring C1A–C6A, the *SHELXL97* similar U_ij_ and rigid bond restraints (*SIMU* and *DELU*) were applied.

### Crystal data

**Table:**

C_17_H_19_NO_3_	*Z* = 6
*M**_r_* = 285.33	*F*(000) = 912
Triclinic, *P*1	*D*_x_ = 1.244 Mg m^−^^3^
Hall symbol: -P 1	Mo *K*α radiation, λ = 0.71073 Å
*a* = 11.565 (5) Å	Cell parameters from 25618 reflections
*b* = 14.010 (4) Å	θ = 1.8–27.6°
*c* = 15.062 (4) Å	µ = 0.09 mm^−^^1^
α = 77.229 (4)°	*T* = 296 K
β = 84.398 (5)°	Plate, yellow
γ = 73.892 (5)°	0.72 × 0.34 × 0.12 mm
*V* = 2284.9 (13) Å^3^	

### Data collection

**Table:**

Stoe IPDSII diffractometer	8981 independent reflections
Radiation source: fine-focus sealed tube	4754 reflections with *I* > 2σ(*I*)
plane graphite	*R*_int_ = 0.042
Detector resolution: 6.67 pixels mm^-1^	θ_max_ = 26.0°, θ_min_ = 1.5°
ω scans	*h* = −14→14
Absorption correction: integration (*X-RED32*; Stoe & Cie, 2002)	*k* = −17→17
*T*_min_ = 0.957, *T*_max_ = 0.992	*l* = −18→17
25618 measured reflections	

### Refinement

**Table:**

Refinement on *F*^2^	Secondary atom site location: difference Fourier map
Least-squares matrix: full	Hydrogen site location: inferred from neighbouring sites
*R*[*F*^2^ > 2σ(*F*^2^)] = 0.062	H atoms treated by a mixture of independent and constrained refinement
*wR*(*F*^2^) = 0.185	*w* = 1/[σ^2^(*F*_o_^2^) + (0.0889*P*)^2^] where *P* = (*F*_o_^2^ + 2*F*_c_^2^)/3
*S* = 1.03	(Δ/σ)_max_ = 0.001
8981 reflections	Δρ_max_ = 0.45 e Å^−^^3^
578 parameters	Δρ_min_ = −0.50 e Å^−^^3^
28 restraints	Extinction correction: *SHELXL*, Fc^*^=kFc[1+0.001xFc^2^λ^3^/sin(2θ)]^-1/4^
Primary atom site location: structure-invariant direct methods	Extinction coefficient: 0.0033 (8)

### Special details

**Table:**

Experimental. 288 frames, detector distance = 100 mm
Geometry. All esds (except the esd in the dihedral angle between two l.s. planes) are estimated using the full covariance matrix. The cell esds are taken into account individually in the estimation of esds in distances, angles and torsion angles; correlations between esds in cell parameters are only used when they are defined by crystal symmetry. An approximate (isotropic) treatment of cell esds is used for estimating esds involving l.s. planes.
Refinement. Refinement of *F*^2^ against ALL reflections. The weighted *R*-factor wR and goodness of fit *S* are based on *F*^2^, conventional *R*-factors *R* are based on *F*, with *F* set to zero for negative *F*^2^. The threshold expression of *F*^2^ > σ(*F*^2^) is used only for calculating *R*-factors(gt) etc. and is not relevant to the choice of reflections for refinement. *R*-factors based on *F*^2^ are statistically about twice as large as those based on *F*, and *R*- factors based on ALL data will be even larger.

### Fractional atomic coordinates and isotropic or equivalent isotropic displacement parameters (Å^2^)

**Table:**

	*x*	*y*	*z*	*U*_iso_*/*U*_eq_	
C1A	0.5986 (3)	0.5390 (2)	0.1914 (2)	0.0739 (7)	
C1B	0.7268 (2)	0.55194 (19)	0.5063 (2)	0.0554 (7)	
C1C	0.8834 (2)	0.53869 (18)	0.85417 (19)	0.0543 (7)	
C2A	0.5104 (3)	0.5079 (3)	0.1644 (3)	0.0886 (8)	
H2A	0.4310	0.5349	0.1825	0.106*	
C2B	0.6716 (2)	0.47435 (19)	0.5147 (2)	0.0585 (7)	
H2B	0.6021	0.4752	0.5516	0.070*	
C2C	0.8125 (3)	0.5360 (2)	0.7868 (2)	0.0622 (7)	
H2C	0.7435	0.5885	0.7721	0.075*	
C3A	0.5357 (3)	0.4367 (3)	0.1102 (3)	0.1025 (9)	
H3A	0.4728	0.4167	0.0924	0.123*	
C3B	0.7178 (2)	0.3959 (2)	0.4692 (2)	0.0599 (7)	
H3B	0.6804	0.3436	0.4769	0.072*	
C3C	0.8417 (2)	0.4568 (2)	0.7406 (2)	0.0618 (7)	
H3C	0.7936	0.4570	0.6945	0.074*	
C4A	0.6511 (3)	0.3947 (3)	0.0818 (3)	0.0847 (8)	
C4B	0.8193 (2)	0.3943 (2)	0.4124 (2)	0.0574 (7)	
C4C	0.9435 (2)	0.37715 (19)	0.76353 (19)	0.0535 (7)	
C5A	0.7385 (3)	0.4257 (3)	0.1086 (3)	0.0851 (8)	
H5A	0.8178	0.3998	0.0896	0.102*	
C5B	0.8760 (3)	0.4707 (2)	0.4033 (2)	0.0701 (8)	
H5B	0.9446	0.4703	0.3655	0.084*	
C5C	1.0144 (2)	0.3798 (2)	0.83097 (19)	0.0590 (7)	
H5C	1.0825	0.3267	0.8468	0.071*	
C6A	0.7132 (3)	0.4959 (2)	0.1644 (3)	0.0821 (8)	
H6A	0.7764	0.5141	0.1840	0.099*	
C6B	0.8299 (3)	0.5482 (2)	0.4510 (2)	0.0676 (8)	
H6B	0.8694	0.5988	0.4456	0.081*	
C6C	0.9857 (2)	0.4600 (2)	0.8752 (2)	0.0593 (7)	
H6C	1.0355	0.4612	0.9195	0.071*	
C7A	0.7786 (3)	0.2916 (2)	−0.0147 (3)	0.0843 (10)	
H71A	0.8379	0.2535	0.0302	0.101*	
H72A	0.8049	0.3496	−0.0489	0.101*	
C7B	0.9671 (3)	0.3015 (3)	0.3177 (3)	0.0918 (11)	
H71B	1.0327	0.2975	0.3551	0.110*	
H72B	0.9617	0.3593	0.2675	0.110*	
C7C	0.9124 (3)	0.2908 (2)	0.6506 (2)	0.0759 (9)	
H71C	0.9030	0.3519	0.6036	0.091*	
H72C	0.8330	0.2849	0.6742	0.091*	
C8A	0.7666 (4)	0.2261 (3)	−0.0774 (3)	0.1043 (13)	
H81A	0.8429	0.2033	−0.1080	0.156*	
H82A	0.7078	0.2643	−0.1216	0.156*	
H83A	0.7415	0.1684	−0.0429	0.156*	
C8B	0.9897 (4)	0.2067 (3)	0.2822 (3)	0.1280 (18)	
H81B	1.0636	0.1975	0.2461	0.192*	
H82B	0.9244	0.2114	0.2452	0.192*	
H83B	0.9956	0.1499	0.3323	0.192*	
C8C	0.9778 (4)	0.1998 (3)	0.6119 (3)	0.1083 (14)	
H81C	0.9328	0.1947	0.5636	0.162*	
H82C	0.9865	0.1398	0.6589	0.162*	
H83C	1.0560	0.2065	0.5884	0.162*	
C9A	0.6362 (3)	0.6629 (2)	0.2591 (2)	0.0634 (8)	
H9A	0.7133	0.6485	0.2324	0.076*	
C9B	0.5829 (3)	0.6563 (2)	0.5894 (2)	0.0667 (8)	
H9B	0.5275	0.6234	0.5787	0.080*	
C9C	0.7487 (3)	0.6580 (2)	0.9270 (2)	0.0641 (8)	
H9C	0.6892	0.6310	0.9134	0.077*	
C10A	0.6030 (2)	0.74064 (19)	0.3135 (2)	0.0572 (7)	
C10B	0.5447 (3)	0.7328 (2)	0.6455 (2)	0.0634 (8)	
C10C	0.7133 (3)	0.74098 (19)	0.9755 (2)	0.0594 (7)	
C11A	0.4868 (2)	0.76687 (18)	0.35280 (19)	0.0552 (7)	
C11B	0.6238 (3)	0.78544 (19)	0.6623 (2)	0.0582 (7)	
C11C	0.7967 (3)	0.78991 (19)	0.99204 (19)	0.0582 (7)	
C12A	0.4578 (3)	0.84182 (19)	0.4053 (2)	0.0595 (7)	
C12B	0.5872 (3)	0.85486 (19)	0.7208 (2)	0.0630 (8)	
C12C	0.7606 (3)	0.8675 (2)	1.0424 (2)	0.0643 (8)	
C13A	0.5422 (3)	0.8900 (2)	0.4160 (2)	0.0685 (8)	
H13A	0.5223	0.9405	0.4502	0.082*	
C13B	0.4725 (3)	0.8708 (2)	0.7595 (2)	0.0776 (9)	
H13B	0.4476	0.9169	0.7980	0.093*	
C13C	0.6418 (3)	0.8957 (2)	1.0720 (2)	0.0747 (9)	
H13C	0.6170	0.9478	1.1044	0.090*	
C14A	0.6569 (3)	0.8645 (2)	0.3763 (2)	0.0770 (9)	
H14A	0.7136	0.8974	0.3844	0.092*	
C14B	0.3935 (3)	0.8197 (3)	0.7422 (3)	0.0922 (11)	
H14B	0.3162	0.8318	0.7688	0.111*	
C14C	0.5590 (3)	0.8483 (2)	1.0546 (3)	0.0814 (10)	
H14C	0.4793	0.8686	1.0754	0.098*	
C15A	0.6866 (3)	0.7913 (2)	0.3256 (2)	0.0704 (8)	
H15A	0.7634	0.7750	0.2986	0.084*	
C15B	0.4284 (3)	0.7512 (3)	0.6860 (3)	0.0877 (11)	
H15B	0.3749	0.7169	0.6747	0.105*	
C15C	0.5929 (3)	0.7720 (2)	1.0072 (2)	0.0738 (9)	
H15C	0.5366	0.7405	0.9958	0.089*	
C16A	0.3035 (3)	0.9448 (2)	0.4879 (3)	0.0798 (10)	
H16D	0.3142	1.0067	0.4482	0.096*	
H16E	0.3498	0.9313	0.5417	0.096*	
C16B	0.6400 (3)	0.9729 (2)	0.7923 (2)	0.0712 (9)	
H16F	0.6199	0.9406	0.8535	0.085*	
H16G	0.5708	1.0275	0.7695	0.085*	
C16C	0.8224 (3)	0.9747 (3)	1.1205 (3)	0.0880 (11)	
H16I	0.7923	0.9417	1.1781	0.106*	
H16H	0.7612	1.0359	1.0969	0.106*	
C17A	0.1739 (4)	0.9550 (3)	0.5138 (4)	0.1238 (18)	
H17D	0.1647	0.8934	0.5533	0.186*	
H17E	0.1293	0.9678	0.4599	0.186*	
H17F	0.1440	1.0104	0.5448	0.186*	
C17B	0.7467 (3)	1.0134 (3)	0.7927 (3)	0.0948 (11)	
H17I	0.7288	1.0619	0.8315	0.142*	
H17G	0.7653	1.0456	0.7318	0.142*	
H17H	0.8146	0.9586	0.8149	0.142*	
C17C	0.9351 (4)	1.0004 (3)	1.1338 (3)	0.1092 (14)	
H17K	0.9190	1.0456	1.1757	0.164*	
H17L	0.9643	1.0327	1.0764	0.164*	
H17M	0.9948	0.9395	1.1579	0.164*	
N1A	0.5638 (2)	0.61332 (16)	0.24631 (17)	0.0616 (6)	
N1B	0.6892 (2)	0.63265 (15)	0.55443 (16)	0.0597 (6)	
N1C	0.8578 (2)	0.62015 (16)	0.90201 (16)	0.0600 (6)	
O1A	0.40193 (19)	0.72146 (16)	0.34247 (17)	0.0770 (7)	
H1A	0.439 (3)	0.681 (3)	0.304 (3)	0.116*	
O1B	0.73590 (19)	0.77207 (15)	0.62429 (16)	0.0741 (6)	
H1B	0.745 (3)	0.722 (3)	0.596 (3)	0.111*	
O1C	0.91309 (17)	0.76443 (15)	0.96280 (15)	0.0680 (6)	
H1C	0.920 (3)	0.708 (3)	0.939 (3)	0.102*	
O2A	0.34239 (19)	0.86173 (15)	0.44148 (16)	0.0773 (6)	
O2B	0.6720 (2)	0.90079 (15)	0.73405 (16)	0.0777 (6)	
O2C	0.8492 (2)	0.90863 (16)	1.05770 (16)	0.0818 (7)	
O3A	0.6664 (2)	0.32439 (19)	0.02880 (18)	0.0941 (8)	
O3B	0.85657 (18)	0.31313 (14)	0.37065 (15)	0.0708 (6)	
O3C	0.98225 (17)	0.29559 (14)	0.72252 (14)	0.0676 (6)	

### Atomic displacement parameters (Å^2^)

**Table:**

	*U*^11^	*U*^22^	*U*^33^	*U*^12^	*U*^13^	*U*^23^
C1A	0.0555 (13)	0.0795 (14)	0.1026 (18)	−0.0235 (11)	0.0102 (12)	−0.0499 (14)
C1B	0.0575 (16)	0.0539 (14)	0.0578 (18)	−0.0138 (12)	−0.0069 (13)	−0.0169 (13)
C1C	0.0546 (16)	0.0519 (14)	0.0589 (18)	−0.0170 (12)	−0.0025 (13)	−0.0128 (13)
C2A	0.0602 (15)	0.1061 (18)	0.118 (2)	−0.0278 (14)	0.0152 (15)	−0.0624 (17)
C2B	0.0525 (15)	0.0630 (15)	0.0641 (19)	−0.0161 (12)	0.0028 (13)	−0.0229 (14)
C2C	0.0617 (17)	0.0576 (15)	0.0618 (19)	−0.0026 (13)	−0.0130 (14)	−0.0128 (14)
C3A	0.0692 (16)	0.131 (2)	0.138 (2)	−0.0391 (15)	0.0168 (16)	−0.0835 (19)
C3B	0.0570 (17)	0.0631 (15)	0.070 (2)	−0.0242 (13)	0.0006 (14)	−0.0251 (14)
C3C	0.0596 (17)	0.0666 (16)	0.0581 (18)	−0.0069 (13)	−0.0157 (13)	−0.0168 (14)
C4A	0.0628 (14)	0.0972 (16)	0.120 (2)	−0.0325 (12)	0.0159 (14)	−0.0687 (16)
C4B	0.0580 (16)	0.0613 (15)	0.0554 (18)	−0.0167 (13)	0.0011 (13)	−0.0173 (13)
C4C	0.0531 (15)	0.0567 (14)	0.0507 (17)	−0.0122 (12)	0.0014 (13)	−0.0150 (13)
C5A	0.0579 (15)	0.0948 (17)	0.122 (2)	−0.0237 (13)	0.0144 (15)	−0.0652 (17)
C5B	0.0719 (19)	0.0650 (17)	0.076 (2)	−0.0281 (15)	0.0196 (16)	−0.0174 (16)
C5C	0.0491 (15)	0.0663 (16)	0.0575 (18)	−0.0029 (12)	−0.0048 (13)	−0.0185 (14)
C6A	0.0573 (14)	0.0926 (17)	0.114 (2)	−0.0230 (13)	0.0047 (14)	−0.0568 (16)
C6B	0.0679 (19)	0.0622 (16)	0.080 (2)	−0.0281 (14)	0.0043 (16)	−0.0194 (15)
C6C	0.0492 (15)	0.0717 (17)	0.0601 (19)	−0.0137 (13)	−0.0076 (13)	−0.0204 (14)
C7A	0.084 (2)	0.079 (2)	0.100 (3)	−0.0289 (17)	0.025 (2)	−0.044 (2)
C7B	0.089 (2)	0.091 (2)	0.096 (3)	−0.0246 (19)	0.038 (2)	−0.038 (2)
C7C	0.077 (2)	0.0804 (19)	0.078 (2)	−0.0150 (16)	−0.0135 (17)	−0.0350 (17)
C8A	0.125 (3)	0.092 (2)	0.110 (3)	−0.034 (2)	0.027 (3)	−0.058 (2)
C8B	0.154 (4)	0.100 (3)	0.135 (4)	−0.036 (3)	0.069 (3)	−0.063 (3)
C8C	0.129 (3)	0.092 (2)	0.113 (3)	−0.009 (2)	−0.028 (3)	−0.056 (2)
C9A	0.0603 (18)	0.0605 (16)	0.070 (2)	−0.0138 (14)	0.0043 (14)	−0.0195 (15)
C9B	0.0636 (19)	0.0622 (16)	0.080 (2)	−0.0107 (14)	−0.0149 (16)	−0.0284 (15)
C9C	0.0609 (18)	0.0610 (16)	0.073 (2)	−0.0163 (13)	−0.0048 (15)	−0.0180 (15)
C10A	0.0605 (17)	0.0529 (14)	0.0601 (19)	−0.0169 (12)	−0.0024 (13)	−0.0130 (13)
C10B	0.0606 (18)	0.0589 (15)	0.072 (2)	−0.0080 (13)	−0.0100 (15)	−0.0214 (14)
C10C	0.0578 (17)	0.0564 (15)	0.0632 (19)	−0.0107 (12)	−0.0028 (13)	−0.0158 (14)
C11A	0.0608 (17)	0.0483 (13)	0.0582 (18)	−0.0193 (12)	0.0024 (13)	−0.0101 (13)
C11B	0.0617 (17)	0.0514 (14)	0.0580 (18)	−0.0066 (12)	−0.0048 (14)	−0.0132 (13)
C11C	0.0587 (17)	0.0582 (15)	0.0558 (18)	−0.0115 (13)	−0.0029 (13)	−0.0120 (13)
C12A	0.0670 (18)	0.0514 (14)	0.0592 (19)	−0.0138 (13)	0.0024 (14)	−0.0136 (13)
C12B	0.0682 (19)	0.0529 (15)	0.069 (2)	−0.0117 (13)	−0.0044 (15)	−0.0198 (14)
C12C	0.073 (2)	0.0589 (15)	0.063 (2)	−0.0143 (14)	−0.0036 (15)	−0.0194 (14)
C13A	0.076 (2)	0.0626 (16)	0.076 (2)	−0.0222 (15)	0.0007 (16)	−0.0293 (16)
C13B	0.079 (2)	0.0725 (19)	0.085 (2)	−0.0134 (17)	0.0087 (18)	−0.0372 (18)
C13C	0.076 (2)	0.0720 (18)	0.077 (2)	−0.0128 (16)	0.0094 (17)	−0.0324 (17)
C14A	0.073 (2)	0.0788 (19)	0.093 (3)	−0.0284 (16)	0.0009 (18)	−0.0381 (19)
C14B	0.067 (2)	0.103 (2)	0.117 (3)	−0.0223 (19)	0.017 (2)	−0.053 (2)
C14C	0.066 (2)	0.084 (2)	0.095 (3)	−0.0134 (17)	0.0122 (18)	−0.033 (2)
C15A	0.0613 (18)	0.0724 (18)	0.083 (2)	−0.0212 (14)	0.0032 (16)	−0.0256 (17)
C15B	0.066 (2)	0.091 (2)	0.120 (3)	−0.0201 (17)	0.000 (2)	−0.053 (2)
C15C	0.0645 (19)	0.0778 (19)	0.083 (2)	−0.0194 (15)	0.0051 (16)	−0.0270 (18)
C16A	0.086 (2)	0.0717 (18)	0.093 (3)	−0.0252 (16)	0.0197 (19)	−0.0437 (18)
C16B	0.091 (2)	0.0589 (16)	0.067 (2)	−0.0121 (15)	−0.0059 (17)	−0.0274 (15)
C16C	0.107 (3)	0.084 (2)	0.089 (3)	−0.0366 (19)	0.012 (2)	−0.040 (2)
C17A	0.091 (3)	0.126 (3)	0.183 (5)	−0.036 (2)	0.047 (3)	−0.103 (3)
C17B	0.108 (3)	0.084 (2)	0.109 (3)	−0.031 (2)	−0.003 (2)	−0.049 (2)
C17C	0.114 (3)	0.115 (3)	0.127 (4)	−0.042 (2)	0.000 (3)	−0.068 (3)
N1A	0.0679 (15)	0.0510 (12)	0.0664 (16)	−0.0144 (11)	0.0041 (12)	−0.0176 (11)
N1B	0.0659 (15)	0.0514 (12)	0.0618 (16)	−0.0100 (11)	−0.0074 (12)	−0.0164 (11)
N1C	0.0619 (15)	0.0575 (12)	0.0635 (16)	−0.0165 (11)	−0.0010 (12)	−0.0180 (11)
O1A	0.0693 (14)	0.0777 (13)	0.1005 (19)	−0.0336 (11)	0.0217 (12)	−0.0451 (12)
O1B	0.0715 (14)	0.0712 (13)	0.0911 (17)	−0.0221 (10)	0.0078 (11)	−0.0404 (12)
O1C	0.0580 (12)	0.0723 (12)	0.0797 (15)	−0.0164 (10)	0.0023 (10)	−0.0309 (11)
O2A	0.0761 (14)	0.0748 (12)	0.0942 (17)	−0.0282 (10)	0.0229 (12)	−0.0452 (12)
O2B	0.0844 (15)	0.0733 (12)	0.0889 (17)	−0.0244 (11)	0.0071 (12)	−0.0445 (12)
O2C	0.0815 (15)	0.0887 (14)	0.0908 (17)	−0.0278 (12)	0.0070 (12)	−0.0481 (13)
O3A	0.0826 (16)	0.1172 (18)	0.113 (2)	−0.0461 (13)	0.0254 (14)	−0.0739 (16)
O3B	0.0683 (13)	0.0693 (11)	0.0820 (15)	−0.0202 (10)	0.0153 (11)	−0.0354 (11)
O3C	0.0641 (12)	0.0707 (12)	0.0700 (14)	−0.0054 (9)	−0.0109 (10)	−0.0307 (11)

### Geometric parameters (Å, °)

**Table:**

C1A—C2A	1.342 (4)	C9B—H9B	0.9300
C1A—C6A	1.358 (4)	C9C—N1C	1.279 (3)
C1A—N1A	1.419 (3)	C9C—C10C	1.451 (4)
C1B—C6B	1.382 (4)	C9C—H9C	0.9300
C1B—C2B	1.384 (4)	C10A—C15A	1.394 (4)
C1B—N1B	1.422 (3)	C10A—C11A	1.399 (4)
C1C—C2C	1.378 (4)	C10B—C11B	1.396 (4)
C1C—C6C	1.382 (4)	C10B—C15B	1.401 (4)
C1C—N1C	1.430 (3)	C10C—C11C	1.397 (4)
C2A—C3A	1.378 (4)	C10C—C15C	1.408 (4)
C2A—H2A	0.9300	C11A—O1A	1.347 (3)
C2B—C3B	1.378 (3)	C11A—C12A	1.401 (4)
C2B—H2B	0.9300	C11B—O1B	1.346 (3)
C2C—C3C	1.385 (4)	C11B—C12B	1.407 (4)
C2C—H2C	0.9300	C11C—O1C	1.349 (3)
C3A—C4A	1.368 (4)	C11C—C12C	1.408 (4)
C3A—H3A	0.9300	C12A—O2A	1.369 (3)
C3B—C4B	1.381 (4)	C12A—C13A	1.371 (4)
C3B—H3B	0.9300	C12B—O2B	1.364 (4)
C3C—C4C	1.389 (4)	C12B—C13B	1.376 (4)
C3C—H3C	0.9300	C12C—O2C	1.367 (4)
C4A—C5A	1.333 (4)	C12C—C13C	1.378 (4)
C4A—O3A	1.364 (4)	C13A—C14A	1.386 (4)
C4B—O3B	1.365 (3)	C13A—H13A	0.9300
C4B—C5B	1.379 (4)	C13B—C14B	1.381 (5)
C4C—O3C	1.364 (3)	C13B—H13B	0.9300
C4C—C5C	1.379 (4)	C13C—C14C	1.381 (5)
C5A—C6A	1.385 (4)	C13C—H13C	0.9300
C5A—H5A	0.9300	C14A—C15A	1.361 (4)
C5B—C6B	1.389 (4)	C14A—H14A	0.9300
C5B—H5B	0.9300	C14B—C15B	1.370 (4)
C5C—C6C	1.377 (4)	C14B—H14B	0.9300
C5C—H5C	0.9300	C14C—C15C	1.362 (4)
C6A—H6A	0.9300	C14C—H14C	0.9300
C6B—H6B	0.9300	C15A—H15A	0.9300
C6C—H6C	0.9300	C15B—H15B	0.9300
C7A—O3A	1.400 (4)	C15C—H15C	0.9300
C7A—C8A	1.495 (5)	C16A—O2A	1.432 (3)
C7A—H71A	0.9700	C16A—C17A	1.487 (5)
C7A—H72A	0.9700	C16A—H16D	0.9700
C7B—O3B	1.428 (4)	C16A—H16E	0.9700
C7B—C8B	1.488 (5)	C16B—O2B	1.431 (3)
C7B—H71B	0.9700	C16B—C17B	1.495 (5)
C7B—H72B	0.9700	C16B—H16F	0.9700
C7C—O3C	1.437 (4)	C16B—H16G	0.9700
C7C—C8C	1.498 (4)	C16C—O2C	1.420 (4)
C7C—H71C	0.9700	C16C—C17C	1.487 (5)
C7C—H72C	0.9700	C16C—H16I	0.9700
C8A—H81A	0.9600	C16C—H16H	0.9700
C8A—H82A	0.9600	C17A—H17D	0.9600
C8A—H83A	0.9600	C17A—H17E	0.9600
C8B—H81B	0.9600	C17A—H17F	0.9600
C8B—H82B	0.9600	C17B—H17I	0.9600
C8B—H83B	0.9600	C17B—H17G	0.9600
C8C—H81C	0.9600	C17B—H17H	0.9600
C8C—H82C	0.9600	C17C—H17K	0.9600
C8C—H83C	0.9600	C17C—H17L	0.9600
C9A—N1A	1.277 (4)	C17C—H17M	0.9600
C9A—C10A	1.452 (4)	O1A—H1A	0.89 (4)
C9A—H9A	0.9300	O1B—H1B	0.88 (4)
C9B—N1B	1.274 (4)	O1C—H1C	0.92 (4)
C9B—C10B	1.456 (4)		
			
C2A—C1A—C6A	117.1 (3)	C11A—C10A—C9A	120.6 (3)
C2A—C1A—N1A	117.0 (3)	C11B—C10B—C15B	119.4 (3)
C6A—C1A—N1A	125.8 (3)	C11B—C10B—C9B	120.9 (3)
C6B—C1B—C2B	117.9 (2)	C15B—C10B—C9B	119.7 (3)
C6B—C1B—N1B	117.2 (3)	C11C—C10C—C15C	119.5 (3)
C2B—C1B—N1B	124.8 (3)	C11C—C10C—C9C	121.2 (3)
C2C—C1C—C6C	118.5 (2)	C15C—C10C—C9C	119.3 (3)
C2C—C1C—N1C	123.2 (2)	O1A—C11A—C10A	122.0 (2)
C6C—C1C—N1C	118.3 (2)	O1A—C11A—C12A	118.7 (2)
C1A—C2A—C3A	121.0 (3)	C10A—C11A—C12A	119.3 (3)
C1A—C2A—H2A	119.5	O1B—C11B—C10B	122.1 (2)
C3A—C2A—H2A	119.5	O1B—C11B—C12B	117.9 (3)
C3B—C2B—C1B	121.0 (3)	C10B—C11B—C12B	120.0 (3)
C3B—C2B—H2B	119.5	O1C—C11C—C10C	122.4 (2)
C1B—C2B—H2B	119.5	O1C—C11C—C12C	117.8 (3)
C1C—C2C—C3C	121.5 (2)	C10C—C11C—C12C	119.8 (3)
C1C—C2C—H2C	119.2	O2A—C12A—C13A	125.1 (2)
C3C—C2C—H2C	119.2	O2A—C12A—C11A	115.0 (3)
C4A—C3A—C2A	121.8 (3)	C13A—C12A—C11A	119.9 (3)
C4A—C3A—H3A	119.1	O2B—C12B—C13B	125.7 (3)
C2A—C3A—H3A	119.1	O2B—C12B—C11B	115.3 (3)
C2B—C3B—C4B	120.5 (3)	C13B—C12B—C11B	119.0 (3)
C2B—C3B—H3B	119.7	O2C—C12C—C13C	125.5 (3)
C4B—C3B—H3B	119.7	O2C—C12C—C11C	115.7 (3)
C2C—C3C—C4C	119.4 (3)	C13C—C12C—C11C	118.9 (3)
C2C—C3C—H3C	120.3	C12A—C13A—C14A	120.7 (3)
C4C—C3C—H3C	120.3	C12A—C13A—H13A	119.6
C5A—C4A—O3A	125.8 (3)	C14A—C13A—H13A	119.6
C5A—C4A—C3A	117.1 (3)	C12B—C13B—C14B	121.2 (3)
O3A—C4A—C3A	117.1 (3)	C12B—C13B—H13B	119.4
O3B—C4B—C5B	125.3 (3)	C14B—C13B—H13B	119.4
O3B—C4B—C3B	115.4 (2)	C12C—C13C—C14C	121.3 (3)
C5B—C4B—C3B	119.3 (3)	C12C—C13C—H13C	119.3
O3C—C4C—C5C	116.0 (2)	C14C—C13C—H13C	119.3
O3C—C4C—C3C	125.0 (3)	C15A—C14A—C13A	120.0 (3)
C5C—C4C—C3C	119.0 (2)	C15A—C14A—H14A	120.0
C4A—C5A—C6A	121.0 (3)	C13A—C14A—H14A	120.0
C4A—C5A—H5A	119.5	C15B—C14B—C13B	120.3 (3)
C6A—C5A—H5A	119.5	C15B—C14B—H14B	119.8
C4B—C5B—C6B	119.6 (3)	C13B—C14B—H14B	119.8
C4B—C5B—H5B	120.2	C15C—C14C—C13C	120.5 (3)
C6B—C5B—H5B	120.2	C15C—C14C—H14C	119.7
C6C—C5C—C4C	120.9 (2)	C13C—C14C—H14C	119.7
C6C—C5C—H5C	119.5	C14A—C15A—C10A	120.7 (3)
C4C—C5C—H5C	119.5	C14A—C15A—H15A	119.6
C1A—C6A—C5A	121.9 (3)	C10A—C15A—H15A	119.6
C1A—C6A—H6A	119.0	C14B—C15B—C10B	120.1 (3)
C5A—C6A—H6A	119.0	C14B—C15B—H15B	119.9
C1B—C6B—C5B	121.6 (3)	C10B—C15B—H15B	119.9
C1B—C6B—H6B	119.2	C14C—C15C—C10C	120.0 (3)
C5B—C6B—H6B	119.2	C14C—C15C—H15C	120.0
C5C—C6C—C1C	120.5 (3)	C10C—C15C—H15C	120.0
C5C—C6C—H6C	119.7	O2A—C16A—C17A	106.8 (3)
C1C—C6C—H6C	119.7	O2A—C16A—H16D	110.4
O3A—C7A—C8A	108.5 (3)	C17A—C16A—H16D	110.4
O3A—C7A—H71A	110.0	O2A—C16A—H16E	110.4
C8A—C7A—H71A	110.0	C17A—C16A—H16E	110.4
O3A—C7A—H72A	110.0	H16D—C16A—H16E	108.6
C8A—C7A—H72A	110.0	O2B—C16B—C17B	107.0 (3)
H71A—C7A—H72A	108.4	O2B—C16B—H16F	110.3
O3B—C7B—C8B	108.1 (3)	C17B—C16B—H16F	110.3
O3B—C7B—H71B	110.1	O2B—C16B—H16G	110.3
C8B—C7B—H71B	110.1	C17B—C16B—H16G	110.3
O3B—C7B—H72B	110.1	H16F—C16B—H16G	108.6
C8B—C7B—H72B	110.1	O2C—C16C—C17C	108.0 (3)
H71B—C7B—H72B	108.4	O2C—C16C—H16I	110.1
O3C—C7C—C8C	107.8 (3)	C17C—C16C—H16I	110.1
O3C—C7C—H71C	110.1	O2C—C16C—H16H	110.1
C8C—C7C—H71C	110.1	C17C—C16C—H16H	110.1
O3C—C7C—H72C	110.1	H16I—C16C—H16H	108.4
C8C—C7C—H72C	110.1	C16A—C17A—H17D	109.5
H71C—C7C—H72C	108.5	C16A—C17A—H17E	109.5
C7A—C8A—H81A	109.5	H17D—C17A—H17E	109.5
C7A—C8A—H82A	109.5	C16A—C17A—H17F	109.5
H81A—C8A—H82A	109.5	H17D—C17A—H17F	109.5
C7A—C8A—H83A	109.5	H17E—C17A—H17F	109.5
H81A—C8A—H83A	109.5	C16B—C17B—H17I	109.5
H82A—C8A—H83A	109.5	C16B—C17B—H17G	109.5
C7B—C8B—H81B	109.5	H17I—C17B—H17G	109.5
C7B—C8B—H82B	109.5	C16B—C17B—H17H	109.5
H81B—C8B—H82B	109.5	H17I—C17B—H17H	109.5
C7B—C8B—H83B	109.5	H17G—C17B—H17H	109.5
H81B—C8B—H83B	109.5	C16C—C17C—H17K	109.5
H82B—C8B—H83B	109.5	C16C—C17C—H17L	109.5
C7C—C8C—H81C	109.5	H17K—C17C—H17L	109.5
C7C—C8C—H82C	109.5	C16C—C17C—H17M	109.5
H81C—C8C—H82C	109.5	H17K—C17C—H17M	109.5
C7C—C8C—H83C	109.5	H17L—C17C—H17M	109.5
H81C—C8C—H83C	109.5	C9A—N1A—C1A	121.3 (2)
H82C—C8C—H83C	109.5	C9B—N1B—C1B	121.3 (3)
N1A—C9A—C10A	122.8 (3)	C9C—N1C—C1C	119.0 (2)
N1A—C9A—H9A	118.6	C11A—O1A—H1A	102 (3)
C10A—C9A—H9A	118.6	C11B—O1B—H1B	105 (3)
N1B—C9B—C10B	122.6 (3)	C11C—O1C—H1C	104 (2)
N1B—C9B—H9B	118.7	C12A—O2A—C16A	117.6 (2)
C10B—C9B—H9B	118.7	C12B—O2B—C16B	118.0 (2)
N1C—C9C—C10C	123.3 (3)	C12C—O2C—C16C	117.7 (3)
N1C—C9C—H9C	118.4	C4A—O3A—C7A	119.4 (3)
C10C—C9C—H9C	118.4	C4B—O3B—C7B	118.4 (2)
C15A—C10A—C11A	119.4 (3)	C4C—O3C—C7C	117.9 (2)
C15A—C10A—C9A	120.0 (3)		
			
C6A—C1A—C2A—C3A	−1.1 (6)	C10B—C11B—C12B—O2B	178.8 (3)
N1A—C1A—C2A—C3A	179.4 (4)	O1B—C11B—C12B—C13B	179.1 (3)
C6B—C1B—C2B—C3B	−0.1 (4)	C10B—C11B—C12B—C13B	−1.1 (4)
N1B—C1B—C2B—C3B	−176.4 (3)	O1C—C11C—C12C—O2C	−0.9 (4)
C6C—C1C—C2C—C3C	0.0 (4)	C10C—C11C—C12C—O2C	177.9 (3)
N1C—C1C—C2C—C3C	178.6 (3)	O1C—C11C—C12C—C13C	179.2 (3)
C1A—C2A—C3A—C4A	0.1 (7)	C10C—C11C—C12C—C13C	−2.1 (4)
C1B—C2B—C3B—C4B	−1.5 (4)	O2A—C12A—C13A—C14A	−179.8 (3)
C1C—C2C—C3C—C4C	1.2 (4)	C11A—C12A—C13A—C14A	−0.9 (5)
C2A—C3A—C4A—C5A	−0.1 (7)	O2B—C12B—C13B—C14B	−179.5 (3)
C2A—C3A—C4A—O3A	179.6 (4)	C11B—C12B—C13B—C14B	0.3 (5)
C2B—C3B—C4B—O3B	−179.7 (3)	O2C—C12C—C13C—C14C	−178.8 (3)
C2B—C3B—C4B—C5B	1.7 (4)	C11C—C12C—C13C—C14C	1.2 (5)
C2C—C3C—C4C—O3C	−179.3 (3)	C12A—C13A—C14A—C15A	0.5 (5)
C2C—C3C—C4C—C5C	−1.1 (4)	C12B—C13B—C14B—C15B	0.3 (6)
O3A—C4A—C5A—C6A	−178.4 (4)	C12C—C13C—C14C—C15C	−0.1 (5)
C3A—C4A—C5A—C6A	1.3 (6)	C13A—C14A—C15A—C10A	−0.6 (5)
O3B—C4B—C5B—C6B	−178.8 (3)	C11A—C10A—C15A—C14A	1.2 (5)
C3B—C4B—C5B—C6B	−0.3 (5)	C9A—C10A—C15A—C14A	179.9 (3)
O3C—C4C—C5C—C6C	178.2 (2)	C13B—C14B—C15B—C10B	−0.2 (6)
C3C—C4C—C5C—C6C	−0.2 (4)	C11B—C10B—C15B—C14B	−0.6 (5)
C2A—C1A—C6A—C5A	2.3 (6)	C9B—C10B—C15B—C14B	177.1 (3)
N1A—C1A—C6A—C5A	−178.2 (3)	C13C—C14C—C15C—C10C	0.0 (5)
C4A—C5A—C6A—C1A	−2.5 (6)	C11C—C10C—C15C—C14C	−0.9 (5)
C2B—C1B—C6B—C5B	1.5 (5)	C9C—C10C—C15C—C14C	178.6 (3)
N1B—C1B—C6B—C5B	178.1 (3)	C10A—C9A—N1A—C1A	178.9 (3)
C4B—C5B—C6B—C1B	−1.3 (5)	C2A—C1A—N1A—C9A	−164.9 (3)
C4C—C5C—C6C—C1C	1.5 (4)	C6A—C1A—N1A—C9A	15.7 (5)
C2C—C1C—C6C—C5C	−1.4 (4)	C10B—C9B—N1B—C1B	174.7 (3)
N1C—C1C—C6C—C5C	−180.0 (2)	C6B—C1B—N1B—C9B	163.4 (3)
N1A—C9A—C10A—C15A	179.4 (3)	C2B—C1B—N1B—C9B	−20.2 (4)
N1A—C9A—C10A—C11A	−1.9 (4)	C10C—C9C—N1C—C1C	−179.8 (3)
N1B—C9B—C10B—C11B	1.1 (5)	C2C—C1C—N1C—C9C	41.6 (4)
N1B—C9B—C10B—C15B	−176.5 (3)	C6C—C1C—N1C—C9C	−139.8 (3)
N1C—C9C—C10C—C11C	4.9 (5)	C13A—C12A—O2A—C16A	5.3 (4)
N1C—C9C—C10C—C15C	−174.6 (3)	C11A—C12A—O2A—C16A	−173.7 (3)
C15A—C10A—C11A—O1A	179.1 (3)	C17A—C16A—O2A—C12A	175.7 (3)
C9A—C10A—C11A—O1A	0.4 (4)	C13B—C12B—O2B—C16B	−0.7 (5)
C15A—C10A—C11A—C12A	−1.6 (4)	C11B—C12B—O2B—C16B	179.5 (3)
C9A—C10A—C11A—C12A	179.7 (3)	C17B—C16B—O2B—C12B	−178.4 (3)
C15B—C10B—C11B—O1B	−179.0 (3)	C13C—C12C—O2C—C16C	10.2 (5)
C9B—C10B—C11B—O1B	3.4 (4)	C11C—C12C—O2C—C16C	−169.8 (3)
C15B—C10B—C11B—C12B	1.2 (4)	C17C—C16C—O2C—C12C	173.7 (3)
C9B—C10B—C11B—C12B	−176.4 (3)	C5A—C4A—O3A—C7A	−11.0 (6)
C15C—C10C—C11C—O1C	−179.4 (3)	C3A—C4A—O3A—C7A	169.3 (4)
C9C—C10C—C11C—O1C	1.2 (4)	C8A—C7A—O3A—C4A	−172.6 (3)
C15C—C10C—C11C—C12C	1.9 (4)	C5B—C4B—O3B—C7B	4.3 (5)
C9C—C10C—C11C—C12C	−177.5 (3)	C3B—C4B—O3B—C7B	−174.2 (3)
O1A—C11A—C12A—O2A	−0.2 (4)	C8B—C7B—O3B—C4B	178.2 (3)
C10A—C11A—C12A—O2A	−179.5 (2)	C5C—C4C—O3C—C7C	−178.8 (3)
O1A—C11A—C12A—C13A	−179.2 (3)	C3C—C4C—O3C—C7C	−0.5 (4)
C10A—C11A—C12A—C13A	1.5 (4)	C8C—C7C—O3C—C4C	175.6 (3)
O1B—C11B—C12B—O2B	−1.0 (4)		

### Hydrogen-bond geometry (Å, °)

**Table:**

*Cg*1, *Cg*2, *Cg*3 and *Cg*4 are the centroids of the C1B–C6B, C1C–C6C, C10A–C15A and C10C–C15C rings, respectively.

**Table:**

*D*—H···*A*	*D*—H	H···*A*	*D*···*A*	*D*—H···*A*
O1A—H1A···N1A	0.89 (4)	1.76 (4)	2.601 (3)	156 (4)
O1B—H1B···N1B	0.88 (4)	1.80 (4)	2.611 (3)	152 (4)
O1C—H1C···N1C	0.92 (4)	1.79 (4)	2.643 (3)	153 (3)
C7C—H71C···Cg1	0.97	2.72	3.5692 (1)	146
C7A—H72A···Cg2^i^	0.97	2.75	3.6644 (1)	157
C16B—H16G···Cg3^ii^	0.97	2.89	3.7935 (1)	156
C16B—H16F···Cg4	0.97	2.78	3.6694 (1)	153

Symmetry codes:  (i) −*x*+1, −*y*+1, −*z*; (ii) *x*, *y*−1, *z*.
